# Towards defining the nuclear proteome

**DOI:** 10.1186/gb-2008-9-1-r15

**Published:** 2008-01-23

**Authors:** J Lynn Fink, Seetha Karunaratne, Amit Mittal, Donald M Gardiner, Nicholas Hamilton, Donna Mahony, Chikatoshi Kai, Harukazu Suzuki, Yosihide Hayashizaki, Rohan D Teasdale

**Affiliations:** 1 ARC Centre of Excellence in Bioinformatics, The University of Queensland, St Lucia, Queensland, 4072, Australia; 2 Institute for Molecular Bioscience, University of Queensland, St Lucia, Queensland, 4072, Australia; 3 Department of Biochemical Engineering and Biotechnology, Indian Institute of Technology, New Delhi, 110016, India; 4 Advanced Computational Modelling Centre, Department of Mathematics, University of Queensland, St Lucia, Queensland, 4072, Australia; 5 Genome Exploration Research, RIKEN Genomic Sciences Center (GSC), RIKEN Yokohama Institute, 1-7-22 Suehiro-cho, Tsurumi-ku, Yokohama, Kanagawa, 230-0045, Japan; 6 Genome Science Laboratory, Discovery Research Institute, RIKEN Wako Institute, 2-1 Hirosawa, Wako, Saitama, 351-0198, Japan

## Abstract

**Background:**

The nucleus is a complex cellular organelle and accurately defining its protein content is essential before any systematic characterization can be considered.

**Results:**

We report direct evidence for 2,568 mammalian proteins within the nuclear proteome: the nuclear subcellular localization of 1,529 proteins based on a high-throughput subcellular localization protocol of full-length proteins and an additional 1,039 proteins for which clear experimental evidence is documented in published literature. This is direct evidence that the nuclear proteome consists of at least 14% of the entire proteome. This dataset was used to evaluate computational approaches designed to identify additional nuclear proteins.

**Conclusion:**

This represents direct experimental evidence that the nuclear proteome consists of at least 14% of the entire proteome. This high-quality nuclear proteome dataset was used to evaluate computational approaches designed to identify additional nuclear proteins. Based on this analysis, researchers can determine the stringency and types of lines of evidence they consider to infer the size and complement of the nuclear proteome.

## Abstract 2

Direct evidence is reported for 2,568 mammalian proteins within the nuclear proteome, consisting of at least 14% of the entire proteome.

## Background

Determination of organellar proteomes - the complement of proteins that reside, even if temporarily, in a specific organelle or subcellular region - is of fundamental importance. Cells are compartmentalized into membrane-bound structures in which specific biochemical processes occur and the function of these proteins is generally highly related to the function of the structure. Once the entire complement of proteins for an individual organelle has been defined, we can begin to systematically understand the molecular networks that control the biological processes occurring within that region.

The nucleus is an extremely complex organelle and is critical to the function of a eukaryotic cell. Therefore, identifying all of the proteins that localize to the nucleus is an important step towards understanding whole cell biology. Now that the genomes of several higher eukaryotes have been fully sequenced, this endeavor is beginning to become feasible. Recently, many high-throughput techniques have taken advantage of the availability of whole genomes and have been employed to localize organellar proteomes. These techniques include mass-spectrometry-based proteomics [1-3], genome-wide open-reading frame green fluorescent protein-tagging [4,5], and gene trap screens [6]. Here, we report the experimental subcellular localization of nuclear proteins in mouse using a high-throughput localization assay based on the expression of myc-tagged proteins. In addition, we extrapolate from this set to estimate the entire nuclear proteome using computational methods. Ultimately, the data presented here form the foundation for future studies into the functional aspects of nuclear biology such that the relationships and interactions between proteins and cellular processes can be explored in more detail.

## Results and discussion

### Subcellular localization assays identify 1,529 nuclear proteins

**Figure 1:**
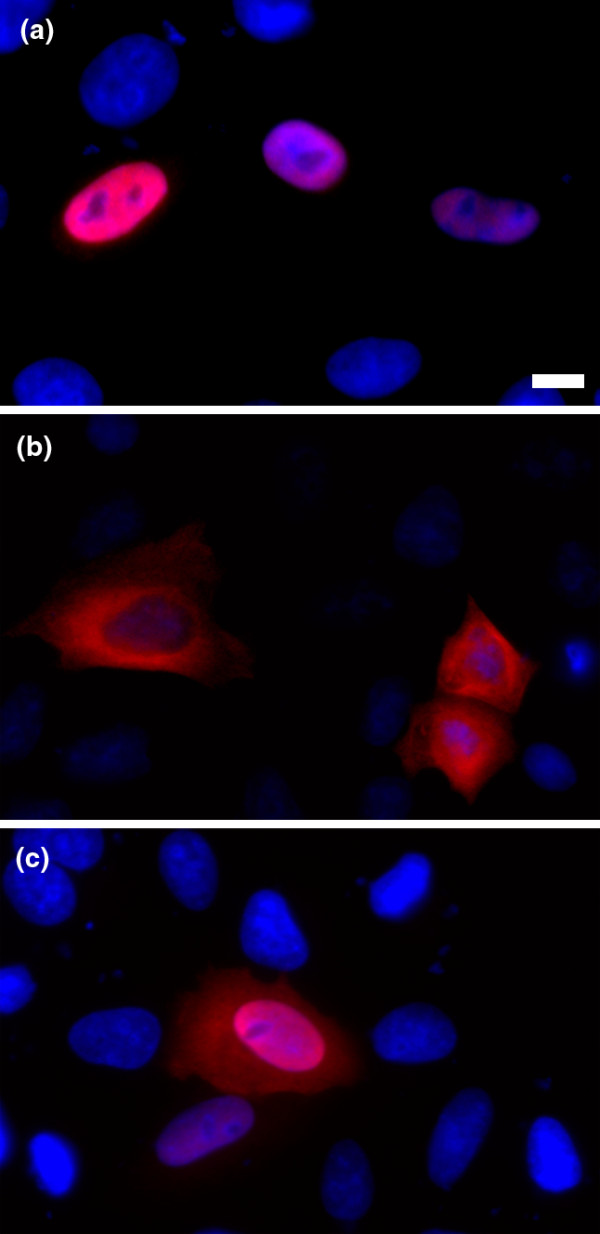
Representative immunofluorescence staining. Amino-terminal myc epitope-tagged expression constructs were generated and expressed in HeLa cells as described previously [15]. The scale bar represents 10 μm. **(a) **IκBα (U36277), a known nuclear protein, localizes to the nucleus. **(b) **Cyln2 (AAH53048) localizes to the cytoplasm. **(c) **Phf21b (AAH67021), a protein with no previous localization data, localizes to both the nucleus and cytoplasm.

As a starting point for accurately defining the nuclear proteome, the proteins that were previously proposed to comprise the entire set of transcription regulators in mouse [7,8] were expressed as full-length, myc-tagged fusion proteins in HeLa cells and immunofluorescence was used to visualize each protein's subcellular location. This dataset of 1,559 was selected because transcription factors are known to act in the nucleus and should represent a large proportion of the nuclear proteome. In total, 1,253 proteins were assayed and localization data were captured for 1,056 proteins. There were 545 proteins that were observed in the nucleus only and 405 were observed in both the nucleus and the cytoplasm, resulting in a total of 950 nuclear proteins (Additional data file 1). Figure 1 shows images of proteins that localize to the nucleus, cytoplasm, or nucleus and cytoplasm and are representative of the images generated for all proteins. All image data have been warehoused in the LOCATE database and can be retrieved from LOCATE Subcellular Localization Database [9-11]. Fifteen proteins were observed to have subcellular localizations in addition to nuclear or cytoplasmic. This small subset predominantly represents inappropriate Gene Ontology (GO) annotations; for example, six are Rab GTPases and should not have been included in the original set of transcriptional regulators. For comparison, in our previous subcellular localization project directed at soluble phosphoregulators [12], only 40% of the proteins examined showed nuclear localization compared to 92% in this study. Importantly, within this study, 20 of 21 proteins reported in the literature to be cytoplasmic only were excluded from the nucleus [12].

**Figure 2:**
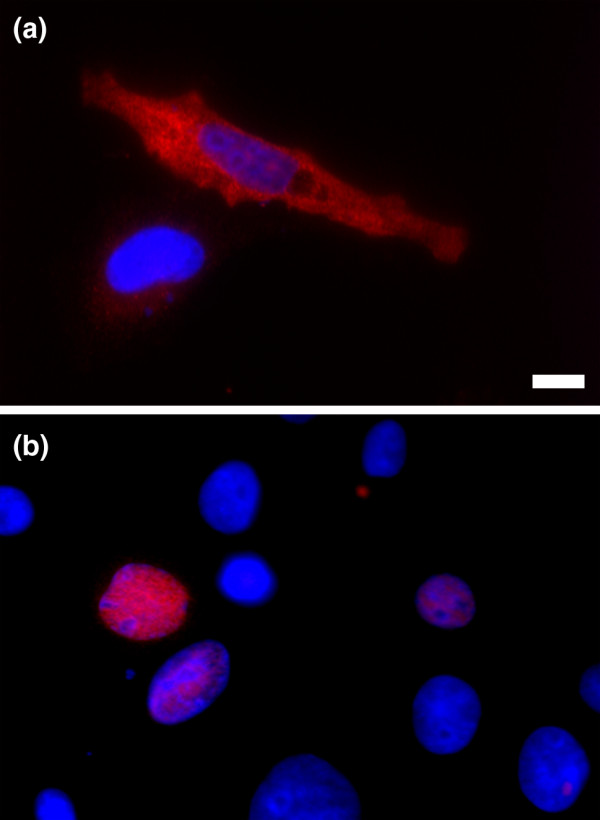
Expression of Trerf1 in two different cell lines. **(a) **Trerf1 (AAH59215) exhibits cytoplasmic localization in HeLa cells. **(b) **When Trerf1 is expressed in MCF7 cells, it localizes to the nucleus. The scale bar represents 10 μm.

It has been observed that organellar proteomes can vary between tissue types [13,14] so proteins that do not exhibit a nuclear localization in HeLa cells may localize to the nucleus in a different cell type. Therefore, the 106 proteins that were observed to reside only in the cytoplasm in HeLa cells were expressed in MCF7 cells. Modulation of the cellular context revealed that 81 of these proteins were localized to the nucleus in MCF7 cells (Figure 2). The small number of 17 proteins, out of the 106 proteins successfully assayed, that did not display a nuclear subcellular localization could be members of the nuclear proteome that require an alternative condition or cellular context not considered in this study. For comparison we selected 200 proteins with a nucleus and cytoplasm subcellular localization in HeLa cells for expression in MCF7 cells. Only ten (five percent) of these proteins displayed a change in subcellular localization, with three classified as nuclear only and seven classified as cytoplasmic only. Eleven failed to yield a protein product that we were able to detect in MCF7 cells. These observations support that, for a fraction of proteins, the cellular context contributes significantly to their subcellular localization.

**Figure 3:**
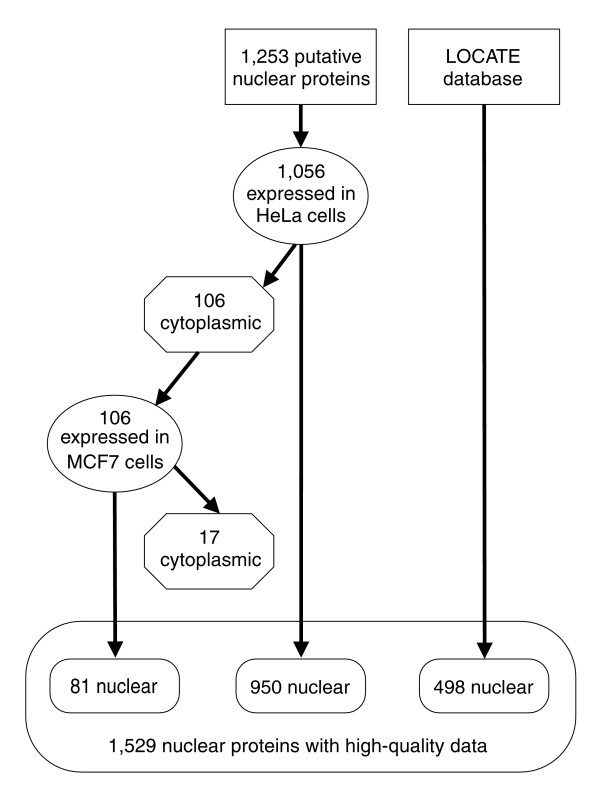
Flowchart describing experimental subcellular localization data acquisition. Experimental data were generated by expressing proteins in HeLa cells and determining their subcellular localization. Proteins that localized to the cytoplasm in HeLa cells were then expressed in MCF7 cells. Proteins reported to localize to the nucleus in the LOCATE database were also included in this dataset. Ultimately, all nuclear proteins were combined, resulting in a set of 1,529 proteins.

In addition to the transcriptional regulator set described above, we have applied the same approach to determine the subcellular localization of additional mouse proteins, including a set of putative type II membrane proteins previously reported [10,15]. As we generate the subcellular localization of these individual proteins the results are deposited within the LOCATE database. To date, we have experimentally observed an additional 498 proteins with a nuclear subcellular localization. Furthermore, within the LOCATE database, published data documenting a protein's subcellular localization are recorded with the original citation. According to the criteria outlined in Fink *et al*. [10], 1,247 proteins have a nuclear subcellular localization reported in the literature. Of the 1,529 nuclear proteins included in our experimental assay, 256 also have associated published literature reporting their subcellular localization, of which 80% of these are nuclear. Given that we have only documented direct evidence for 1,247 mammalian proteins to be part of the nuclear proteome, the identification of 1,529 novel nuclear proteins represents a significant increase in the number of proteins associated with this organelle (Figure 3). While the original transcriptional regulator set was biased towards transcription factors, only 60% of the final set of proteins experimentally localized are transcription factors (that is, have the GO nucleic acid binding annotation).

Other projects that document the subcellular localization of proteins based on direct evidence include the Nuclear Protein Database [16], which includes 1,227 proteins within the nuclear proteome based predominantly on literature and gene-trap experiments [6], and an alternative mammalian subcellular localization project [17] that reports 132 nuclear proteins in the LifeDB database [5,18]. UniProt v9.0 and MGI v3.5 (excluding proteins annotated as a result of reviewed computational analysis) contain evidence for 1,316 and 1,641 nuclear mouse proteins, respectively. However, a proportion of these proteins is not directly supported but is inferred based on a number of approaches.

We selected to exploit cell line models to define the nuclear proteome as they contain all the fundamental machinery to correctly target exogenously expressed proteins to the nucleus. Any tissue specific compartmentalization or regulation of subcellular localization will predominantly involve expression of additional binding proteins or regulatory pathways that function via post-translational modification, the majority of which are not likely to be functional within our subcellular localization assay. To determine if proteins examined in our subcellular localization assay with restricted expression are targeted differentially in our cell model we examined the GNF mouse tissue atlas data [19]. We considered a protein was 'restricted' if it was expressed in 1-40 tissue samples and 'broad' if expressed in 80-128 of the tissue samples. For the 553 proteins within the restricted class, 47% were nucleus only and 38% were nuclear/cytoplasmic, and within the 419 proteins broadly expressed, 38% were nucleus only and 47% were nuclear/cytoplasmic. These observations suggest that our subcellular localization assay captures a protein's intrinsic potential to localize into the nucleus regardless of its expression profile.

Combining the data from the subcellular localization assays with the set of nuclear proteins for which experimental evidence is documented in peer-reviewed literature, we created a high-quality nuclear proteome set of 2,568 proteins, termed NUCPROT. This dataset represents 14% of all mouse genes and contains protein products that have been experimentally confirmed to localize, at least in part, to the nucleus. Of particular importance are 532 proteins annotated as being a hypothetical protein [20] or as having an unknown function; the subcellular localization data reported here provide the first clues to their cellular roles.

### Estimation of the size of the mouse nuclear proteome

Generation of the high-quality set of validated nuclear proteins, NUCPROT, enabled the critical evaluation of the distinct computational approaches developed to define the nuclear proteome. The following methods were used to predict additional putative nuclear proteins. Firstly, a number of computational methods have been developed as 'broad-based subcellular localization predictors' able to predict the subcellular localization of a protein to one or more of several specified locations. To further estimate the extent of the nuclear proteome, we selected five of these methods using criteria described previously [21] and applied them to the entire mouse proteome. We then extracted the proteins predicted to localize to the nucleus by each method. Secondly, it has been observed that the subcellular localization of proteins tends to be conserved across species and within protein families. 'Homology-based methods' to identify proteins related to the NUCPROT set will identify related proteins across species or within mouse. For example, a recent proteomics approach found that the human homolog to a yeast protein that localizes to the nucleolus has nearly a 90% chance of localizing to the same organelle [22]. Thirdly, another method of inferring nuclear localization of a protein is the prediction of nuclear localization signals (NLS). The NLS is a short peptide sequence that functions as a sorting signal to facilitate the import of a protein into the nucleus.

**Figure 4:**
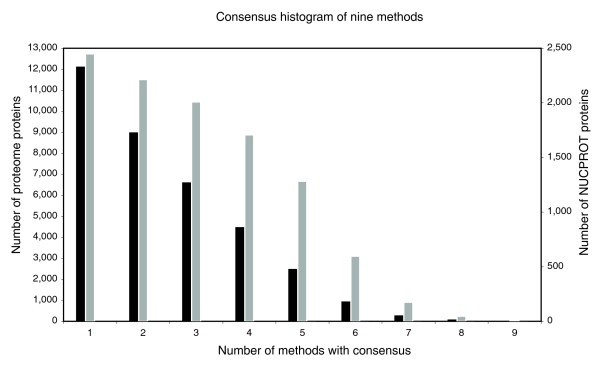
Consensus histogram of nine localization methods. The numbers of proteins that were predicted to be nuclear by each of nine methods are shown as bars. We selected proteins that were predicted to be nuclear by at least four methods. The black bars represent the proteins from the entire mouse proteome while the gray bars represent proteins from the NUCPROT set.

Details of the computational approaches applied are described in Materials and methods. Table 1 summarizes the results of each of these approaches applied to the high-quality set of nuclear proteins, NUCPROT, and also to the entire mouse proteome as defined [10,11,20]. Figure 4 shows the frequency with which each protein was classified as nuclear by these methods.

**Table 1:** Results from computational approaches predicting nuclear proteome membership

Method	NUCPROT proteins classified as 'nuclear'	Accuracy	RIKEN proteome proteins classified as 'nuclear'
CELLO	2,125	78%	9,122 (47%)
pTARGET	1,706	63%	5,953 (30%)
Proteome Analyst	1,803	66%	4,084 (21%)
WoLF PSORT	1,909	70%	7,172 (37%)
MultiLoc	1,561	57%	5,137 (26%)
			
Yeast homology (E < 10^-4^)	218	8.0%	2,031 (8.0%)
Yeast homology (E < 10^-30^)	47	1.7%	691 (3.5%)
Mouse homology	430	16%	766 (3.9%)
			
Nucleo	857	32%	987 (5.0%)

For the subcellular localization prediction programs, proteins were considered to be incorrectly classified as 'not nuclear' if the method's top-ranked localization call was not 'nucleus' but the protein was in our high-quality dataset; proteins were considered to be correctly classified as 'nuclear' if the method's top-ranked localization call was 'nuclear' and the protein was in our high-quality dataset.

Using the homology approach, we compared mouse proteins that are homologous to yeast proteins that were determined to have a nuclear localization by a proteome-wide analysis of protein localization [4]. Using a stringent approach, we found 691 mouse proteins not already included in NUCPROT while a more permissive approach found 2,031 proteins. We also employed the homology approach to select other proteins within the mouse proteome that may be nuclear by inferring homologs to the NUCPROT dataset and found 766 additional homologous mouse proteins. Using the subcellular localization predictors, we found that between 4,084 and 9,122 proteins were predicted to localize to the nucleus and the NLS predictor, Nucleo, predicted that 987 proteins contain a signal.

Each of these methods has its own unique aspect and will likely define part of the nuclear proteome the others will fail to detect. Application of the inferred nuclear proteome will vary, so inclusion based on subsets of the nine computational approaches will be required. Sixty-two percent of the entire mouse proteome, including the NUCPROT set, was predicted by at least one method to be nuclear, resulting in a maximal nuclear proteome. However, a reasonable conservative estimate would include those proteins that were predicted by four out of nine methods, a threshold that included over half of the NUCPROT set. This results in a nuclear proteome of 28% of the mouse proteome, or 5,422 proteins. We term this set of proteins the NUCPROT+4-inferred dataset.

We can compare our NUCPROT+4-inferred dataset to other nuclear proteome estimates. Firstly, based on the combination of any subcellular localization annotation from all major protein databases, 3,848 mouse proteins have been annotated as nuclear within the subcellular localization database LOCATE [10,11]. While these annotations are frequently not supported by direct experimental evidence, they represent an estimate of the current nuclear proteome. For comparison, 91% of these proteins are included in the NUCPROT+4-inferred dataset. Secondly, of the 5,422 NUCPROT+4-inferred dataset, only 49% of these are annotated with the 'cellular component' GO term 'nucleus' in the MGI database.

## Conclusion

Obtaining a fully defined nuclear proteome with each protein having been experimentally localized to the nucleus will ultimately require the determination of the subcellular localization of the entire mammalian proteome and, based on our observations, across a range of cell types. At the moment this is beyond peptide sequencing proteomic approaches and will likely require high-throughput epitope-tagged cell expression approaches like that commenced in this study. Our initial survey puts us on the path towards defining the nuclear proteome with direct evidence that 14% of the mouse proteome contributes to the nuclear proteome. Having defined individual proteins as contributing to the nuclear proteome, then clearly the next stage is to delineate the nucleocytoplasmic trafficking pathways [23] that contribute to each individual nuclear protein's distribution and how their subcellular localization is regulated under distinct cellular conditions.

## Materials and methods

### Dataset

The mouse proteome dataset selected for this analysis was the Isoform Protein Sequence set created by the RIKEN FANTOM3 Consortium from novel and public protein coding transcripts and has been described previously [7,10,24]. This dataset was supplemented with additional sequences reported to belong to the set of mouse transcriptional regulators [8] and consists of a total of 19,562 transcriptional units. Within the text we do not consider the multiple isoforms generated from a single protein coding transcriptional unit.

### Protein subcellular localization

The methods published by Aturaliaya *et al*. [15] were followed, unless stated otherwise, for making expression constructs, transfection into cells, and immunolabeling and image capture. Expression constructs of cDNA clones with an amino-terminal myc epitope were generated and transfected into HeLa and/or MCF7 cells cultured at 60-70% confluency. Expression of proteins was detected 24 hours after transfection by immunolabeling with monoclonal anti-myc antibody (Cell Signaling Technology, Inc., Boston, MA, USA). Cell monolayers were treated with DAPI to label the nuclei. Images were captured on an Olympus AX-70 upright fluorescence microscope. Protein localization data were classified into 'nuclear', 'cytoplasmic', and 'nuclear and cytoplasmic' based on the predominant type of expression in each transfected sample.

### Automated image classification

Subcellular localizations were inferred from the images by both an expert curator and the automated image classification program ASPiC [25]. ASPiC is a fully automated system that assigns a subcellular location to an image. It selects, masks and crops cells within each image, using a corresponding DAPI image to localize the nucleus, generates image statistics, and produces an automated classification for each cropped cell image using a support vector machine. If, for a given protein, there are multiple cells with multiple classifications, a vote is taken to give an overall classification. Average image intensities and areas of the nuclear and non-nuclear regions are also recorded for each cropped cell. Three out of 1,608 images classified by ASPiC were assigned locations that conflicted with the location assigned by a human curator; these conflicts were resolved during a manual review by a second expert curator.

### Computational predictions

Predictions using programs that predict subcellular localization to multiple cellular locations were performed as described previously [21]. Briefly, publicly available programs that predicted localization to at least nine major locations (nucleus, cytoplasm, mitochondrion, extracellular region, plasma membrane, Golgi apparatus, endoplasmic reticulum, peroxisome, and lysosome) and could accept large sequence batches were used to predict locations for all proteins encoded by the mouse transcriptome; these were CELLO [26], WoLF PSORT [27], MultiLOC [28], Proteome Analyst [29,30], and pTARGET [31].

Nuclear localization signals were predicted by predictNLS [32,33], NucPred [34], and Nucleo [35]. NucPred and Nucleo predictions at or above 0.8 were considered to be positive.

### Homology inference

Homologs were inferred by performing a BLAST search [36] of the entire mouse proteome with itself and with nuclear yeast proteins from the Yeast GFP Fusion Localization Database [4]. BLAST hits that did not have sequence coverage of 50% or more were discarded from further analysis. An optimal E-value threshold for selecting homologs was determined by maximizing the number of positives while minimizing the number of negatives using the set of high-confidence nuclear mouse proteins as a set of true positives and the remainder of the mouse proteome as a set of true negatives. The optimal E-value threshold was 10^-140 ^for mouse proteins and 10^-4 ^for yeast proteins. An additional, more stringent E-value threshold of 10^-30 ^was selected for the yeast proteins based on a previous study of computed gene homology [37].

## Abbreviations

GO, Gene Ontology; NLS, nuclear localization signals.

## Authors' contributions

SK, AM, DG and DM participated in the generation of experimental cell biology data. JLF performed the bioinformatics studies, integration of the data and drafted the manuscript. NH developed and implemented the image analysis protocols. CK, HS and YH designed and generated all the transcript templates used in this study. RDT conceived of the study, and participated in its design and coordination. All authors read and approved the final manuscript.

## Additional data files

The following additional data are available. Additional data file 1 is a table listing the subcellular localization data generated in this project and the nuclear proteome, NUCPROT+4-inferred. The table includes a list of proteins in the nuclear proteome, the experimentally determined subcellular location, and data annotating the degree of confidence in their membership. Proteins are referred to by Entrez Gene_ID, GenBank accession number, and b RIKEN representative protein set ID [20].

## Supplementary Material

Additional data file 1 The table includes a list of proteins in the nuclear proteome, the experimentally determined subcellular location, and data annotating the degree of confidence in their membership. Proteins are referred to by Entrez Gene_ID, GenBank accession number, and b RIKEN representative protein set ID [20]. Click here for file
